# Establishment of Hematological Reference Values among Healthy Adults in Bamenda, North West Region of Cameroon

**DOI:** 10.1155/2021/6690926

**Published:** 2021-02-24

**Authors:** Nfor Omarine Nlinwe, Yunika Larissa Kumenyuy, Che Precious Funwi

**Affiliations:** The University of Bamenda, Faculty of Health Sciences, Department of Medical Laboratory Science, P.O. Box 39 Bambili, Bamenda, North West Region, Cameroon

## Abstract

The use of the reference range of values of a laboratory test is highly significant in diagnostic accuracy. However, race and ethnic variations may affect the safe use of reference ranges from a different setting/population. Because the establishment of reference ranges for the Cameroonian population will possibly improve the quality of health care, this study was designed to establish hematological reference ranges among healthy adults in Bamenda, North West region of Cameroon. This was a cross-sectional study carried out within the period of five months from February 2020 to June 2020, at the Bamenda Regional Hospital. A total of 350 (139 females and 211 males) study participants who met the inclusion criteria were included in the study. The Urit 3300 autoanalyzer (Urit Medical Electronic (Group) Co., Ltd, Guilin, China) was used to analyze the hematological parameters. The general health questionnaire for donors, for verification of reference range study and laboratory tests, was used for data collection. Descriptive statistics were used to calculate reference ranges, means, and medians at 95% confidence intervals. Maximum and minimum reference ranges were computed at 97.5th and 2.5th percentiles. The nonparametric test (Mann–Whitney test) was used to determine the significance of the difference in hematological values between the male and female groups. Three (MID%, LYM#, and MID#) out of the 19 hematological parameters were verified, while sixteen (WBC, LYM%, GRAN%, GRAN#, RBC, HGB, HCT%, MCV, MCH, MCHC, RDW_CV, RDW_SD, PLT, MPV, PDW, and PCT%) were established. The currently used reference intervals do not represent the population of the North West region. Therefore, other regional hospitals in Cameroon should establish reference intervals applicable to their respective regions.

## 1. Introduction

A reference range of values of a laboratory test is the basis for results interpretation and patient management; therefore, it is highly significant in diagnostic accuracy [1]. Due to variation in topographical, social, and health status, it is unsafe to use reference ranges from a different setting/population (race and ethnicity) [2]. Other causes of variation in reference ranges include body mass index, sex, age, genetics, altitude, and environmental factors like pathogens [2–4]. Studies have shown differences in hematological parameters among different populations [5–8]. For example, differences in normal hematological values have been reported between subjects with African and non-African lineage [6, 7]. Hence to ensure accurate diagnosis and clinical research the ethnicity of study participants should be considered [8]. In Africa, clinical trials of preventive interventions for particularly malaria, HIV, and tuberculosis are common. The use of inappropriate reference intervals for trial screening may cause excessive elimination of prospective participants. Cameroon has a population with diverse ethnic backgrounds and reference values among medical laboratories across the nation [9]. The establishment of reference ranges for the Cameroonian population will possibly improve the quality of health care and clinical trials. Moreover, the Clinical and Laboratory Standard Institute (CLSI) endorses the establishment of reference ranges by each laboratory [4]. Until now, no reference ranges have been established for the laboratory of the Bamenda Regional Hospital. Therefore, this study was designed to establish hematological reference ranges among healthy adults in Bamenda, North West region of Cameroon.

## 2. Method

### 2.1. Study Site and Population

The study was carried out at the Bamenda Regional Hospital. Bamenda is situated at a height of about 1258 meters above the sea level, and it is located along 10.15 longitude and 5.96 latitudes. Bamenda being the regional headquarter for the North West region is one of the ten regional headquarters in Cameroon. The North West region has seven divisions: Boyo, Bui, Donga-Mantung, Mezam, Menchum, Momo, and Ngoketunjia divisions. There are many ethnic groups in the North West region, including immigrants from other regions and countries like Nigeria. The main ethnic groups of the North West regions are of Tikar origin, which includes the Tikari, Moghamo, Fulani, and Widikum [10]. Bamenda has an estimated 337,036 inhabitants [11].

### 2.2. Ethical Considerations

Ethical approval and written informed consent for this study were obtained from the Ethical Review Committee of the University of Bamenda (2020/0115H/UBa/IRB: 2020/0149H/UBa/IRB) and all the study participants, respectively.

### 2.3. Study Participants/Study Period

This was a cross-sectional study carried out within the period of five months from February 2020 to June 2020. According to the Clinical and Laboratory Standards Institute Guidance Document C28A2A [12], a minimum sample size of 300 (120 males and 120 females) was targeted for this study. The study participants were consecutively recruited from apparently healthy voluntary nonremunerated blood donors between the ages of 18 and 60 years, who gave their consent to be part of the study. A total of 350 (139 females and 211 males) study participants who met the inclusion criteria were included in the study.

### 2.4. Exclusion Criteria

All pregnant/lactating/menstruating women, those who were underweight (body mass index of ≤18.5), had lost ˃10% body weight in the past 6 months, had jaundice in the past 12 months, had a blood transfusion, sickle cell anemia, on any medication, and/or had an unexplained fever in the past 3 months were excluded from the study. Also, all those who tested positive for hepatitis B virus (HBV), hepatitis C virus (HCV), malaria (*Plasmodium falciparum* antigen histidine-rich protein 2 rapid diagnostic test), *Treponema pallidum* hemagglutination (TPHA), and human immunodeficiency virus 1/2 antibodies (HIV 1/2 antibodies) were excluded from the study.

### 2.5. Laboratory Analysis

Screening for confounding factors was performed for each study participant [12]. 5 mL of venous blood was collected into the K3EDTA test tubes. Using whole blood/serum, laboratory tests were carried out for the diagnosis of malaria (PfHRP2), HBV (HBsAg RDTs), HCV (anti-HCV antibody detection), TPHA, HIV 1/2 antibodies, and the determination of ABO blood group and genotype [13, 14].

### 2.6. Blood Cell Count Analysis

The validated hematology analyzer the Urit 3300 autoanalyser (Urit Medical Electronic (Group) Co., Ltd, Guilin, China) was used. Validation of the analyzer was done to confirm that the level and procedure of measurement is satisfactory and correct and that the calibration was appropriately done. The detection principle of the analyzer is electrical impedance (for WBC/RBC/PLT) and photoelectric colorimetry (for HGB). The hematology analyzer was validated by checking its performance characteristics, which was compared against the manufacture's claim as per the package insert. These characteristics included precision, accuracy, linearity, reportable range—analytical measurement range (AMR) and clinical reportable range (CRR), carryover, sensitivity, and specificity [4]. Following the manufacturer's instructions, the analyzer was calibrated and maintained, and appropriate internal quality controls were done daily.

### 2.7. Data Analysis

The general health questionnaire for donors for verification of reference range study was used to collect demographic data. Laboratory tests were done to collect data based on the analysis of hematological parameters and confounding factors. Hematological parameters for 40 participants were initially analyzed. For each parameter, if ˃10% of the values were out of the manufacturer's reference range, it was considered unverified [12]. If otherwise, hematological parameters were considered verified. Categorical data were presented as frequencies and percentages. Descriptive statistics were used to calculate reference ranges, means, and medians at 95% confidence intervals. Maximum and minimum reference ranges were computed at 97.5th and 2.5th percentiles. The nonparametric test (Mann–Whitney test) was used to determine the significance of the difference in hematological values between the male and female groups. GraphPad Prism version 9.0.1 and Microsoft Excel were used for the data analysis.

### 2.8. Definition of the Analyzed Hematological Parameters

WBC: white blood cell, LYM%: lymphocyte%, MID%: midsized cells (monocytes)%, GRAN%: granulocyte%, LYM#: lymphocyte#, MID#: midsized cells (monocytes)#, GRAN#: granulocyte#, RBC: red blood cell, HGB: hemoglobin, HCT%: hematocrit%, MCV: mean corpuscular volume, MCH: mean cell hemoglobin, MCHC: mean cell hemoglobin concentration, RDW_CV: red blood cell volume distribution width-CV, RDW_SD: red blood cell volume distribution width-SD, PLT: platelet, MPV: mean platelet volume, PDW: platelet distribution width, PCT%: plateletcrit%, CV: coefficient of variation, SD: standard deviation, #: number, and %: percentage.

## 3. Results and Discussion

The ≥18 to 30 years age group was the most (75.14%) represented, while the ˃50 to 60 years age group was the least represented. Most (43.71%) of the study participants were from Mezam Division. 55.14% of the study participants had the O blood group, and 96% were Rhesus positive. The male : female sex ratio of the study participants was 1.52 : 1 (Table 1).

For both the female and male groups, three (MID%, LYM#, and MID#) out of the 19 hematological parameters were verified. For the female group, the out-of-range percentage varied from 0% (MID#) to 95% (RDW_SD). However, the out-of-range percentage for the male group ranged from 0% (MID#) to 82.5% (MPV) (Table 2). The combined out-of-range percentage for the hematological parameters extended from 0% (MID#) to 95% (RDW_SD) for the current study but extended from 3.5% (RBC) to 46.7% (MCV) in Asmara, Eritrea [15]. For both sexes, group reference values were lower for WBC, GRAN%, GRAN#, MCV, and RDW_SD and higher for Lym%, RBC, HGB, MCHC, PLT, MPV, and PCT% parameters, compared to the manufacturer's reference values. The reference values for HCT% were higher for females. However, for males, the HCT% reference values exceeded both limits of the manufacturer's reference range. Similarly, in both sex groups, the reference values of MCH, RDW_CV, and PDW exceeded both limits of the manufacturer's reference range.

The hematological profile of the current study population differs from what was reported for the central province of Iran (Southwestern Asia) [16]. The median values of HGB, WBC, and GRAN% values were lower, but the median values of PLT and LYM% were higher, compared to that reported for Iran. It was established that compared to European-Americans, African-Americans have lower values of HGB, HCT%, RBC, PLT, MCV, WBC, GRAN#, and LYM% [6, 7]. Similar to the African-Americans, reference values for the current study were lower for WBC, GRAN#, and MCV, but contrarily, the values were higher for HGB, RBC, PLT, MCV, and LYM%, compared to the standard. However, compared to subjects from the USA, subjects from Southern and Eastern Africa had higher HCT%, HGB levels, WBC, and neutrophil counts [7]. Like in the current study, significantly lower MCV and MCH were reported among the Kenyan population [17]. Comparing the current results with that reported for Southern and Eastern Africa, although HGB and HCT% levels were also higher, WBC and GRAN# were rather lower. This indicates discrepancies among the reference values of African-Americans, Southern, and Eastern Africans and Cameroonians of the North West region. In Nigeria, based on locally established reference values, only 10% of study participants were classified abnormal compared to more than 40% who were classified by US DAIDS [18] and hence the need for locally established reference values.

Twelve out of the 19 (63.16%) hematological parameters were significantly different between the males and females (see Tables 3 and 4). The difference in three (RBC, HGB, and HCT%) out of the 12 (25%) parameters agrees with the differences indicated in the manufacturer's reference values. The median values of RBC, HGB, and HCT% were significantly higher in the male group. Similarly, the median values of RBC, HGB, and HCT% were significantly higher in the male group in a study carried out five years ago, in the same study area as the current study [19]. However, the difference in 9 (MID%, GRAN%, MID#, GRAN#, MCV, MCH, PLT, MPV, and PCT%) out of the 12 (75%) parameters disagree with the uniformity (among male and female values) indicated in the manufacturer's reference values. The median values of MID%, MID#, MCV, MCH, and MPV were significantly higher in the male group, whereas the median values of GRAN%, GRAN#, PLT, and PCT% were significantly higher in the female group. The median values of GRAN# and PLT were also significantly higher in the female group in the previous study of the same study area [19]. The median hemoglobin concentration was 15.2 g/L in men and 12.9 g/L in women (*p* < 0.001).

Studies have equally reported significantly higher values of reticulocyte counts, RBC counts, HGB, and HCT% in adult males compared to the females [1, 2, 9, 17, 20–22]. B12 deficiency and comorbidities were more common in elderly men [23], while in a population-based study among healthy Ugandan population, RBC counts, HGB, and HCT% were higher in adult males than females [2]. Similarly, RBC counts, HGB, and HCT% were higher in adult males than females in three regions in Ghana [20]. Median HGB concentration was equally higher among the men than the women in Yaounde, Cameroon [9]. HGB levels were lower, while platelet counts were higher in females among the healthy adult population in Kenya [17]. From many other studies, PLT counts were found to be higher across different female age groups, as compared to the males [1, 15, 17, 21]. Among normal Nigerian adults, platelets were found to be significantly higher among females than males [1]. Likewise in North West Ethiopia, platelets were found to be significantly higher among females than males [21]. In fact, in Asmara Eritrea, except platelet counts which was higher among the females, all the elevated hematological analytes were found to be higher in males [15]. This is also in line with findings from the Central Region of Cameroon [9]. Generally, the trend on the differences in hematological parameters between males and females is similar across most African countries.

WBC and neutrophil counts were also found to be significantly lower among black women when four indigenous groups based in the United Kingdom were studied [24]. In North West Ethiopia [21], Yaounde Cameroon [9], and in the current study (Bamenda Cameroon), WBC counts were higher among the females. Also, LYM#, NEUT#, and MID# counts were all higher among females in North West Ethiopia [21]. This is however contrary to what was reported in Asmara Eritrea, where values of all hematological analytes (except PLT count) were found to be higher in males than females [15] (see Table 5). Topographical and ethnic variances could have accounted for these differences.

There are many confounders when analyzing hematological parameters in the general population. Some inconsistencies in the values of hematological parameters may be caused by confounding factors such as cold agglutinin disease [25, 26]. For example, initial hemogram test results were significantly affected by cold agglutinin disease, causing incorrect results [26]. However, those with cold agglutinin disease-associated symptoms like jaundice were excluded from the current study. Seasonal variation was also demonstrated in the levels of white blood cell counts and neutrophils in a large population-based study [27]. And even after having adjusted for possible confounders, higher depression and anxiety were shown to cause increase RDW and WBC levels [28]. Plasma markers of inflammation were also shown to have a strong independent association with increased levels of RDW [29]. WBC levels in the current study were rather generally lower than the manufacturer's reference range. Although, the neutrophil-lymphocyte ratio is an inflammatory marker for major adverse cardiac events [30], due to stringent exclusion criteria set for the current study, participants with major adverse cardiac events could not have been enrolled into the study.

### 3.1. Limitations

The study was conducted using blood donors. Although blood donors are generally considered as healthy, they are prone to developing iron deficiency, especially in repeat donors with insufficient iron intake. Although iron deficiency does not necessarily translate into deviations of hematology parameters, the possibility cannot be excluded either.

## 4. Conclusion

Only three (MID%, LYM#, and MID#) out of the 19 hematological parameters considered in this study were verified. Sixteen (WBC, LYM%, GRAN%, GRAN#, RBC, HGB, HCT%, MCV, MCH, MCHC, RDW_CV, RDW_SD, PLT, MPV, PDW, and PCT%) hematological parameters have been established, demonstrating that the currently used reference intervals do not represent the population. Based on the commendation by the Clinical and Laboratory Standard Institute (CLSI) that reference ranges be established by each laboratory, published reference intervals (literature and manufacturer) should not be used without local verification. Therefore, to ensure adequate clinical care and perhaps successful subsequent clinical trials in Cameroon, further studies to establish hematological parameters for other regions in Cameroon are recommended.

## Figures and Tables

**Table 1:** Sociodemographic factors, blood group, and body mass index of the study participants.

Variables		Male (%)	Female (%)	Total (%)
Age (years)	≥18–30	142 (67.3)	121 (87.1)	263 (75.1)
	>30–40	50 (23.7)	17 (12.2)	67 (19.1)
	>40–50	14 (6.6)	0	14 (4)
	>50–60	5 (2.4)	1 (0.7)	6 (1.7)

Division of origin	Mezam	98 (46.5)	55 (39.6)	153 (43.7)
	Bui	24 (11.4)	31 (22.3)	55 (15.7)
	Momo	27 (12.8)	14 (10.1)	41 (11.7)
	Donga-Mantung	14 (6.6)	5 (3.4)	19 (5.4)
	Ngonketunjia	14 (6.6)	6 (4.3)	20 (5.7)
	Menchum	10 (4.7)	5 (3.6)	15 (4.3)
	Boyo	7 (3.3)	2 (1.4)	9 (2.6)
	Non-North west	17 (8.1)	21 (15.1)	38 (10.9)

Blood group	O	111 (52.6)	82 (59)	193 (55.1)
	A	46 (21.8)	28 (20.1)	74 (21.1)
	B	46 (21.8)	25 (18)	71 (20.3)
	AB	8 (3.8)	4 (2.9)	12 (3.4)

Rhesus factor	Positive	205 (97.2)	131 (94.2)	336 (96)
	Negative	6 (2.8)	8 (5.8)	14 (4)

Body mass index	Underweight (≤8.5)	0	0	0
	Normal weight (>8.5–24.99)	102 (48.3)	59 (42.5)	161 (46)
	Overweight (>24.99–29.99)	81 (38.4)	44 (31.7)	125 (35.7)
	Obesity (>29.99)	28 (13.3)	36 (25.9)	64 (18.3)

Total (%)		211 (60.3)	139 (39.7)	350

Sources: compiled by authors.

**Table 2:** Obtained reference intervals and manufacturer's reference intervals of hematological parameters.

Parameter	Unit	RI for males, *N* = 211	Manufacturer's RI for males	Out of range (%)	RI for females, *N* = 139	Manufacturer's RI for females	Out of range (%)
WBC	10^3^/*μ*L	2.86–8.54	4–10	18	3.0–10	4–10	30
LYM%	%	27.89–62.42	20–40	73	27.2–62.38	20–40	75
MID%	%	6–17.58	1–15	5^*∗*^	5.05–13.42	1–15	10^*∗*^
GRAN%	%	29.09–59.31	50–70	77.5	31.02–68.43	50–70	65
LYM#	10^3^/*μ*L	1.2–4.5	1–4.1	2.5^*∗*^	1.04–4.86	1–4.1	7.5^*∗*^
MID#	10^3^/*μ*L	0.2–1.1	0.1–1.8	0^*∗*^	0.2–1.3	0.1–1.8	0^*∗*^
GRAN#	10^3^/*μ*L	1.2–4.4	2–7.8	37.5	1.2–5.2	2–7.8	30
RBC	10^6^/*μ*L	4.4–7.0	4–5	80	3.6–7.3	3.5–5	25
HGB	g/dL	12.1–18.2	12–16	17.5	8.8–19.24	11–15	22.5
HCT%	%	35.1–51.69	42–49	45	25.1–55.6	37–43	67.5
MCV	fL	63.21–89.81	82–92	57.5	57.41–90.1	82–92	50
MCH	pg	22.4–31.9	27–31	30	20.09–33.46	27–31	50
MCHC	g/dL	32.1–37.9	32–36	17.5	31.79–38.66	32–36	22.5
RDW_CV	%	10.4–15.4	11.5–14.5	37.5	10.6–16.51	11.5–14.5	25
RDW_SD	fL	24.8–43.7	37–54	80	24.17–44.3	37–54	95
PLT	10^3^/*μ*L	104.4–338.8	100–300	12.5	102–656.9	100–300	37.5
MPV	fL	8.33–14.1	7.4–10.4	82.5	8.1–14.78	7.4–10.4	82.5
PDW	fL	9.07–17.04	10.0–14	37.5	8.6–16.96	10–14	42.5
PCT%	%	0.11–0.39	0.1–0.28	30	0.1–0.76	0.1–0.28	47.5

Sources: compiled by authors. ^*∗*^Out of range percentage for verified hematological parameter.

**Table 3:** Calculated hematological (RBC and RBC indices) reference intervals for the screened population.

Parameter (unit)	Sex	*N*	Mean	95% CI for mean	Median	95% CI for median	Range	2.5th–97.5th%	*P* value (gender)
RBC (10^6^/*μ*L)	Female Male	139 211	4.74 5.4	4.61–4.87 5.33–5.48	4.68 5.35	4.56–4.77 5.3–5.43	3.23–8.77 3.91–7.31	3.57–7.23 4.41–6.99	<0.0001^*∗*^

HGB (g/dL)	Female Male	139 211	12.94 15.34	12.62–13.26 14.99–15.69	12.9 15.2	12.6–13.1 15–15.4	7.6–20 11.5–47.4	8.8–19.2 12.13–18.15	<0.0001^*∗*^

HCT% (%)	Female Male	139 211	39.92 43.39	35.85–37.99 42.85–43.93	36.6 43.3	35.6–37.3 42.4–44	33.3–69.6 32.7–56.4	25.1–55.6 35.1–51.69	<0.0001^*∗*^

MCV (fL)	Female Male	139 211	78.2 80.23	76.96–79.45 79.29–81.17	78.8 80.2	78–80.3 79.7–82.4	54.5–92 35.4–93.4	57.45–90.1 63.21–89.81	0.0073^*∗*^

MCH (pg)	Female Male	139 211	27.41 28.14	26.96–27.87 27.84–28.43	27.6 28.2	27.3–28 28–28.7	19.5–35.2 20.3–34.6	20.1–33.45 22.38–31.92	0.0030^*∗*^

MCHC (g/dL)	Combined Female Male	350 139 211	35.24 35.2 35.23	34.92–35.39 34.95–35.54 34.73–35.74	35.2 35.2 35.1	35–35.2 35–35.4 34.8–35.2	28.5–85.1 28.5–42 31.5–85.1	32–38.65 31.79–39.53 32.09–37.92	0.4309

RDW_CV (%)	Combined Female Male	350 139 211	12.7 12.85 12.6	12.54–12.85 12.57–13.13 12.42–12.77	12.45 12.4 12.5	12.3–12.6 12.3–12.8 12.2–12.7	10–19.3 10.2–19.3 10–17.3	10.5–15.82 10.6–16.5 10.43–15.37	0.4467

RDW_SD (fL)	Combined Female Male	350 139 211	33.31 33.15 33.41	32.71–33.9 32.16–34.15 32.66–34.16	33.9 33.9 33.9	33–34.7 29.7–34.8 33–34.7	22.2–50.6 23.1–45.4 22.2–50.6	24.8–44.3 24.2–44.3 24.8–43.68	0.6062

Sources: compiled by authors.  ^*∗*^Significant *P* values.

**Table 4:** Calculated hematological (WBC and platelet) reference interval for the screened population.

Parameter (unit)	Sex	*N*	Mean	95% CI for mean	Median	95% CI for median	Range	2.5th–97.5th%	*P* value (gender)
WBC (10^3^/*μ*L)	Combined Female Male	350 139 211	5.29 5.47 5.17	5.13–5.45 5.18–5.77 4.98–5.36	5 5.1 4.9	4.9–5.2 4.9–5.5 4.8–5.2	1.7–12.6 1.7–12.6 2.6–10.8	3–9.42 3.3–10.25 2.86–8.54	0.0877

LYM% (%)	Combined Female Male	350 139 211	44.15 43.63 44.53	43.24–45.06 42.19–45.07 43.35–45.71	43.95 43.55 44.4	42.7–44.8 42.1–44.8 42.7–45.5	4.5–70.4 14.9–70.4 4.5–64.8	27.59–61.66 27.2–62.38 27.89–62.42	0.3727

MID% (%)	Female Male	139 211	8.33 10.17	7.98–8.69 9.75–10.58	8.1 9.6	7.8–8.4 9-10	0.2–16.9 4.3–24.4	5.05–13.42 6–17.58	<0.0001^*∗*^

GRAN% (%)	Female Male	139 211	48.03 45.02	46.51–49.54 43.94–46.1	47.6 45.2	46.8–49 43.5–47.3	15.7–78.2 13.1–70.2	31.02–68.43 29.09–59.31	0.0034^*∗*^

LYM# (10^3^/*μ*L)	Combined Female Male	350 139 211	2.35 2.39 2.32	2.26–2.43 2.24–2.54 2.21–2.42	2.2 2.2 2.1	2.1–2.3 2.1–2.3 2.1-2.2	0.5–5.6 0.5–5.5 0.9–5.6	1.2–4.7 1.04–4.86 1.2–4.48	0.4327

MID# (10^3^/*μ*L)	Female Male	139 211	0.46 0.52	0.43–0.5 0.49–0.55	0.4 0.5	0.4-0.4 0.4-0.5	0.2–1.4 0.2–1.3	0.2–1.3 0.2–1.07	0.0007^*∗*^

GRAN# (10^3^/*μ*L)	Female Male	139 211	2.6 2.33	2.45–2.76 2.23–2.43	2.4 2.2	2.3–2.5 2.1–2.3	0.7–5.8 1.8–5.1	1.2–5.2 1.2–4.37	0.0034^*∗*^

PLT (10^3^/*μ*L)	Combined Female Male	139 211	294 222.8	274.8–313.2 214.8–230.9	281 224	263–307 217–231	64–870 29–453	102–656 104.4–338.8	<0.0001^*∗*^

MPV (fL)	Combined Female Male	139 211	10.99 11.98	10.67–11.32 11.65–12.31	11 12.5	10.6–11.8 12.2–12.6	7.7–15.6 7.8–36.6	8.1–14.75 8.33–14.1	<0.0001^*∗*^

PDW (fL)	Combined Female Male	350 139 211	12.78 12.59 12.91	12.56–13.01 12.2–12.99 12.64–13.18	12.6 12.2 12.9	12.2–13.3 11.8–14 12.2–13.3	6.8–23 8.2–23 6.8–18.3	8.91–16.81 8.6–16.95 9.09–16.99	0.0623

PCT% (%)	Female Male	139 211	0.31 0.27	0.29–0.33 0.24–0.31	0.29 0.26	0.27–0.31 0.25–0.28	0.09–0.93 0.02–3.6	0.11–0.75 0.11–0.39	<0.0001^*∗*^

Sources: compiled by authors.  ^*∗*^Significant *P* values.

**Table 5:** Comparison of obtained reference intervals and reference intervals in Yaounde and other African countries.

Parameter (unit)	Sex	Obtained RI	Man.'s RI	Out of range (%)	Bamenda Cameroon (2015)	Yaounde Cameroon	Eritrea	Tanzania	Ghana	Ethiopia
RBC (10^6^/*μ*L)	Female Male	3.57–7.23 4.41–6.99	3.5–5 4-5	25 80	4.12–5.48 4.42–6.13	3.4–5.3 4–5.9	4–5.7 4.2–6.07	3.84–5.59 4.41–6.27	3.09–5.3 3.79–5.96	3.45–6.25 3.53–6.93

HGB (g/dL)	Female Male	8.8–19.2 12.13–18.15	11–15 12–16	22.5 17.5	10.9–14.5 12.4–16.4	9.89–13.7 11–16	12.5–17.6 12.6–17.8	11.1–15.7 13.7–17.7	8.8–14.4 11.3–16.4	11–16.7 15.3–18

HCT% (%)	Female Male	25.1–55.6 35.1–51.69	37–43 42–49	67.5 45	32.8–44.2 37–49.8	29.7–42 34.6–47.61	37.9–52 40.5–55	36.2–46.8 40.2–53.7	26.4–45 33.2–50.2	32.1–56.6 36.2–58.6

MCV (fL)	Combined Female Male	— 57.45–90.1 63.21–89.81	82–92 — —	— 50 57.5	— 71.6–92.7 68.2–93.3	— 72–96 70–97	— 85.5–100 85.7–100	— 77.7–97.9 76.4–98.8	— 73–96 70–98	85–100 — —

MCH (pg)	Combined Female Male	— 20.1–33.45 22.38–31.92	27–31 — —	— 50 30	— 23.1–30.5 22.4–31.6	— 23.22–31.2 22.8–33.4	— 26.5–32.6 28–33	— 24.2–33.1 23.1–33.2	— 22.3–33.6 22.7–33.5	— 25.8–32.8 26.6–33.3

MCHC (g/dL)	Combined Female Male	— 31.79–39.53 32.09–37.92	32–36 — —	— 22.5 17.5	— 31.2–34.4 31.8–34.6	— 30–34.2 29.9–35.3	— 30–33.7 30.4–33.7	— 30.4–34.8 30.6–35.1	— 30.4–36.5 22.3–33.6	— 28.5–34.4 29.5–34.4

WBC (10^3^/*μ*L)	Combined Female Male	3–9.4 3.3–10.25 2.86–8.54	4–10 — —	— 30 18	3.2–8.3 3.6–8.3 3.0–8.2	— 2.8–6.7 2.6–6.81	— 3.3–8.9 3.7–9.3	— 3.2–8 2.8–7.9	— 3.4–9.3 3.5–9.2	3.2–8.8 — —

PLT (10^3^/*μ*L)	Combined Female Male	102.6–459.5 102–656 104.4–338.8	100–300 — —	— 37.5 12.5	142–354 148–367 140–346	— 143–369 133–339	— 145.4–351.6 128.4–318.4	— 151–425 147–356	— 89–403 88–352	128–432

Sources: compiled by authors and [9, 15, 19]. Man.'s RI = manufacturer's reference interval.

## Data Availability

The data used to support the findings of this study are available from the corresponding author on request.
